# Metabolic Changes in the Visual Cortex of Binocular Blindness Macaque Monkeys: A Proton Magnetic Resonance Spectroscopy Study

**DOI:** 10.1371/journal.pone.0080073

**Published:** 2013-11-05

**Authors:** Lingjie Wu, Zuohua Tang, Xinghuai Sun, Xiaoyuan Feng, Wen Qian, Jie Wang, Lixin Jin

**Affiliations:** 1 Department of Radiology, Eye and ENT Hospital of Shanghai Medical School, Fudan University, Shanghai, China; 2 Department of Ophthalmology, Eye and ENT Hospital, State Key Laboratory of Medical Neurobiology, Institutes of Brain Science, Fudan University, Shanghai, China; 3 Department of Radiology, Huashan Hospital of Shanghai Medical School, Fudan University, Shanghai, China; 4 Siemens Ltd. Healthcare Sector, Shanghai, China; Institute of Automation, Chinese Academy of Sciences, China

## Abstract

**Purpose:**

To evaluate proton magnetic resonance spectroscopy (^1^H-MRS) in a study of cross-modal plasticity in the visual cortex of binocular blindness macaque monkeys.

**Materials and Methods:**

Four healthy neonatal macaque monkeys were randomly divided into 2 groups, with 2 in each group. Optic nerve transection was performed in both monkeys in the experimental group (group B) to obtain binocular blindness. Two healthy macaque monkeys served as a control group (group A). After sixteen months post-procedure, ^1^H-MRS was performed in the visual cortex of all monkeys. We compared the peak areas of NAA, Cr, Cho, Glx and Ins and the ratios of NAA/Cr, Cho/Cr, Glx/Cr and Ins/Cr of each monkey in group B with group A.

**Results:**

The peak area of NAA and the NAA/Cr ratio in the visual cortex of monkey 4 in group B were found to be dramatically decreased, the peak area of NAA slightly decreased and the NAA/Cr ratio clearly decreased in visual cortex of monkey 3 in group B than those in group A. The peak area of Ins and the Ins/Cr ratio in the visual cortex of monkey 4 in group B slightly increased. The peak area of Cho and the Cho/Cr ratio in the visual cortex of all monkeys in group B dramatically increased compared with group A. The peak area of Glx in the visual cortex of all monkeys in group B slightly increased compared with group A.

**Conclusions:**

^1^H-MRS could detect biochemical and metabolic changes in the visual cortex and therefore this technique can be used to provide valuable information for investigating the mechanisms of cross-modal plasticity of binocular blindness in a macaque monkey model.

## Introduction

The loss of any one modality can lead to signal generation in the deprived cortical area from inputs originating from other modalities, illustrating the remarkable capacity of the cerebral cortex for plasticity [1]. This phenomenon, in which a change in one sensory system alters the development of the remaining sensory systems in different stage of life, is called cross-modal plasticity [2]. For instance, congenital or early visual loss subjects acquire important anatomical and functional reorganization of the visually-deprived cortex that becomes activated by a variety of non-visual stimuli, such as touch, audition and olfaction [3]. Therefore, it seems that the visual cortex of the blind is capable of adapting to accommodate these non-visual inputs through cross-modal plasticity [1]. However, the exact mechanisms underlying cross-modal plasticity in the visual cortex of vision loss subjects is a major question in the research field. Although some previous studies have reported morphological, functional, behavioral and biochemical changes, which have been used to explain cellular and synaptic plasticity mechanisms that uphold anatomical and functional adaptations in the cerebral cortex, the biochemical and metabolic changes in the visual cortex after loss of sight are not clear [4-7].

In vivo ^1^H-MRS is a powerful tool for noninvasively investigating metabolism in a specific region of the brain. There have even been reports about biochemical and metabolic changes detected in the visual cortex with this approach [8-10]. Chan et al[10] reported a reduction in choline (Cho) in the visual cortex of rats with glaucoma, which may be due to the dysfunction of the cholinergic system in the visual system caused by progressive visual loss. In addition, reduction of NAA and taurine (Tau) levels in the visual cortex contralateral to the enucleated eye of neonatal monocular enucleated rats was reported by Chow et al [8]. Such results may reflect a reduction of neurons and overall neuronal activity in the visual cortex of monocular blind rats. Furthermore, ^1^H-MRS also has been applied to humans with acquired blindness at different ages to reveal a significant increase of myo-inositol (Ins) in the visual cortex, which suggests that glial cells may play an active role in the reorganization of the cortex in response to visual deprivation [9]. These studies suggest that measurements of metabolites in the visual cortex can provide valuable information to study the metabolic changes associated with cross-modal plasticity following progressive vision loss (glaucoma), monocular blindness in rats and late-stage blindness in humans; however, no studies have been conducted on neonatal binocular blind subjects.

Therefore, our study utilized macaque monkeys to establish a neonatal binocular blindness model to investigate the mechanisms of cross-modal plasticity in the visual cortex of binocular blind subjects by detecting biochemical and metabolic changes in vivo using ^1^H-MRS at 3T. Such alterations in the metabolism of the visual cortex maybe valuable to future studies of biochemistry, changes in metabolism and cortical reorganization associated with the adaptive reactions caused by visual loss or other sensory deprivations. To our knowledge, we are the first to report the investigation of biochemical and metabolic changes in the visual cortex of early binocular blindness macaque monkeys in vivo using ^1^H-MRS.

## Materials and Methods

### Ethics statement

This study was carried out in strict accordance with the recommendations in the Guide for the Care and Use of Laboratory Animals of Fudan University and according to local and international ethical guidelines. The protocol was approved by the Committee on the Ethics of Animal Experiments of the Eye & ENT Hospital. All macaque monkeys were housed in an air-conditioned room with an ambient temperature of 21–25°C, a relative humidity of 40%–60% and a 12 h light/dark cycle. During the light period, all monkeys were free to roam the air-conditioned room, which had swings, erect wood and toys such as basketballs and puzzles. During the dark period, all monkeys were housed in individual 100 cm×70 cm×80 cm cages with visual, auditory and olfactory contacts with their peers. In addition, all monkeys were provided a balanced diet including fresh fruits, vegetables, cereals, bread and vitamins twice daily, in addition to water that was freely available. Furthermore, veterinarians that were skilled in the healthcare and maintenance of macaque monkeys supervised their health. Prior to optic nerve transection of monkeys, they were anesthetized with a 0.3ml ketamine-xylazine mixture. Postoperatively, each monkey received cefotaxime (75 mg/kg body weight) and an analgesic, diclofenac sodium (1 mg/kg body weight), twice daily for 5 days. No monkey was sacrificed during the study.

### Animal preparation and experimental protocol

Healthy neonatal macaque monkeys (n=4) were randomly divided into 2 groups, with 2 in each group. In this study, optic nerve transection was performed in bilateral eyes of each monkey in the experimental group (Group B, monkey 3 and monkey 4) at postnatal day 40 with 0.3ml ketamine-xylazine mixture anaesthesia to obtain binocular blindness. Through an incision in the bitamporal conjunctiva, we transected the superior oblique and bluntly dissected the fascia tissue around eyeball until the optic nerve was clearly exposed, then we transected the retrobulbar optic nerve at the site of 0.5mm away from the sclera. In addition, a small section of optic nerve was removed from the surgical eye to ensure the optic nerve was completely transected. Finally, the eyeball of the macaque was reset into the orbit again, sutured the incision in the conjunctiva and swabbed Tobrex oculentum within the conjunctival sac. This operation is believed to result in less drastic morphological changes and surgical trauma than the binocular enucleated model. Separate, healthy macaque monkeys were kept as a control group (Group A, monkey 1 and monkey 2). Sixteen months after operation, ^1^H-MRS was performed in the visual cortex of all monkeys.

### Magnetic resonance spectroscopy

^1^H-MRS was performed using a Siemens–Magnetom Verio 3.0T scanner (Germany), with a gradient of 45mT/m and maximum switchover rate of 200T/m/s. Anesthetized macaque monkeys were placed in the knee coil in a supine position with their heads in the central location. Thin sheets were used to keep monkeys warm at approximately 38°C.

For all macaque monkeys, single voxel ^1^H-MRS was performed in the same MR unit in the visual cortex of the occipital lobe. The volume of interest (VOI) for acquiring ^1^H-MRS was chosen based on criteria including the largest possible voxel within the visual cortex. To preselect the VOI, a fast spin-echo (FSE) in three planes was used to acquire high resolution anatomical images for position the voxel of ^1^H-MRS along standard anatomical orientation. The location of 1H-MRS was achieved in sagittal T2WI images. The VOI was positioned to include the bilateral visual cortex as far as possible while avoiding the inclusion of skull, fat and air sinus. Six saturated zones were placed around the VOI to reduce the effect of surrounding tissues. Shimming and tuning were achieved with automated procedures before scanning. Water signals were suppressed with selective water signal inversion. ^1^H-MRS scanning started when the FWHM (full width at half maximum) between 47 and 53 Hz, the water suppression (WS) ≧98%. Spectra were recorded with the following parameters: TR=2000ms, TE=30ms, thickness=15mm, averages=180, FOV=140×140mm^2^, NEX=2, Voxel=18×20×15 mm^3^.

### Data post-processing

Spectra analysis was performed on a Siemens MR post-processing work station (Siemens–Magnetom, Verio, 3.0T MR, Germany). Five experienced operators performed all MRS post-processing independently. Resonance peaks in the spectrum was determined as NAA (2.02ppm), Cr (3.02ppm), Cho (3.2ppm), Ins (3.5-3.65ppm) and Glx (2.1-2.5ppm and 3.74ppm). Furthermore the peak area of the metabolites was measured automatically by calculating the integral area under the curve of the metabolites using the MR post-processing work station, which represented their relative concentrations. Concentration ratios of NAA, Cho, Ins and Glx were calculated in reference to Cr, which was considered an internal standard for the examination.

We compared the relative concentrations of the metabolites mentioned above and the NAA/Cr, Cho/Cr, Glx/Cr and Ins/Cr ratios in the visual cortex of each monkey in group B with those in group A.

## Results

**Figure 1:**
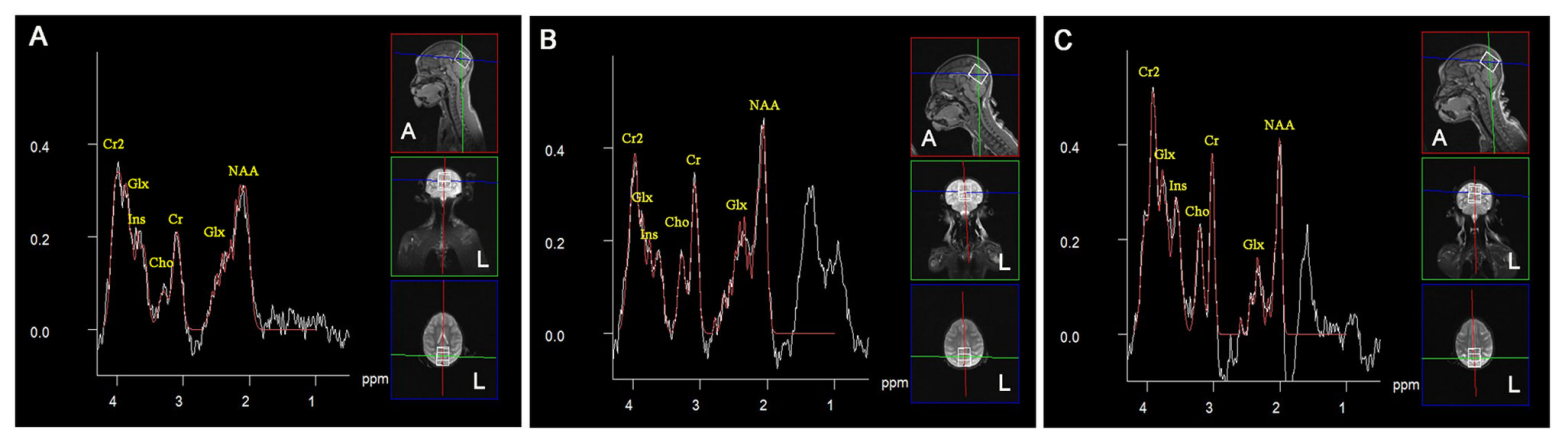
Biochemical and metabolic spectra in the visual cortex. ^1^H-MRS spectra were obtained from the visual cortex of normal control macaque monkeys in group A (A) and sixteen months after binocular visual deprivation in monkey 3 (B) and monkey 4 (C) in group B. The peak area of NAA and the NAA/Cr ratio in the visual cortex of monkey 4 in group B were found to be dramatically decreased, while the peak area of NAA slightly decreased and the NAA/Cr ratio clearly decreased in visual cortex of monkey 3 in group B than those in group A. The Ins/Cr ratio of monkey 4 slightly increased. The peak area of Cho and the Cho/Cr ratio in the visual cortex of all monkeys in group B dramatically increased. The peak area of Glx in the visual cortices of all monkeys in group B slightly increased compared with group A. NAA: N-acetyl aspartate, Cho: choline, Cr: creatine, Glx: glutamate + glutamine, Ins: myo-inositol.

The peak areas of NAA, Cho, Ins, Cr and Glx in the visual cortex of all monkeys are summarized in Table 1. Concentration ratios of NAA/Cr, Cho/Cr, Glx/Cr and Ins/Cr in the visual cortex of all monkeys are displayed in Table 2. Note that if the peak area or the ratio of each metabolite increased or decreased by 5%-15%, 16%-29% or more than 30% compared with the group A, we ruled that the differences were slight, clear and dramatic, respectively. The peak area of NAA and the NAA/Cr ratio in the visual cortex of monkey 4 in group B were found to be dramatically decreased compared with the corresponding mean value of group A, meanwhile the peak area of NAA slightly decreased and the NAA/Cr ratio clearly decreased in visual cortex of monkey 3 in group B than those in group A (Figure 1, Table 1 and 2). The peak area of Ins in the visual cortex of monkey 4 clearly increased, while the Ins/Cr ratio slightly increased, whereas the peak area of Ins and the Ins/Cr ratio of monkey 3 clearly decreased. The peak area of Cho and the Cho/Cr ratio in the visual cortex of all monkeys in group B dramatically increased than group A (Figure 1, Table 1 and 2). The peak area of Glx in the visual cortices of all monkeys in group B slightly increased, while the Glx/Cr ratio slightly decreased (Figure 1, Table 1 and 2).

**Table 1:** The peak areas of NAA, Cho, Glx, Cr and Ins in the visual cortex.

	**Group A**			**Group B**		
	Monkey 1	Monkey 2	Mean	Monkey3	Monkey4	Mean
**NAA**	6.71	7.50	7.11	6.66 ↓	4.32 ↓↓↓	5.49↓↓
**Cr**	6.32	7.39	6.86	7.82 ↑	7.45 ↑	7.64 ↑
**Ins**	10.71	11.16	10.94	8.50 ↓↓	12.66 ↑↑	10.58 ↓
**Cho**	1.77	1.89	1.83	2.85↑↑↑	3.71↑↑↑	3.28 ↑↑↑
**Glx**	9.85	9.54	9.70	10.29 ↑	10.34 ↑	10.32 ↑

Note: Group A: normal control macaques, Group B: binocular blind macaques; ↑/↓ increased/decreased 5%-15%; ↑↑/↓↓ increased/decreased 16%-29%; ↑↑↑/↓↓↓ increased/decreased more than 30%.

**Table 2:** The ratios of NAA/Cr, Cho/Cr, Glx/Cr and Ins/Cr in the visual cortex.

	Group A			Group B		
	Monkey 1	Monkey 2	Mean	Monkey 3	Monkey 4	Mean
**NAA/Cr**	1.06	1.01	1.04	0.85↓↓	0.58 ↓↓↓	0.72↓↓
**Ins/Cr**	1.69	1.51	1.60	1.09↓↓	1.70 ↑	1.40 ↓
**Cho/Cr**	0.28	0.26	0.27	0.36 ↑↑↑	0.50 ↑↑↑	0.43 ↑↑↑
**Glx/Cr**	1.56	1.29	1.43	1.32 ↓	1.39 ↓	1.36 ↓

Note: Group A: normal control macaques, Group B: binocular blind macaques; ↑/↓ increased/decreased 5%-15%; ↑↑/↓↓ increased/decreased 16%-29%; ↑↑↑/↓↓↓ increased/decreased more than 30%.

## Discussion

Visual deprivation during early life could cause the visual cortex to be activated by other sensory systems through cross-modal plasticity, which has been demonstrated in previous experiments [4,11]. There are two hypotheses regarding the mechanisms of cross-modal plasticity in the visual cortex after visual deprivation [1]. The first hypothesis proposes that early deprived visual cortical circuits can be rewired and/or cross-wired with other modalities and formatted with new permanent connections from other sensory receptors. The second hypothesis suggests that physically present but functionally silent connections between the deprived visual cortex and spared cortices could be activated and/or enhanced following vision loss. However, the biochemical and metabolic changes associated with cross-modal plasticity in the visual cortex following vision loss are not clear. Hence, in our study, we investigated the biochemical and metabolic changes in the visual cortex of macaque monkeys after binocular blindness to determine their effects on cross-modal plasticity in models of vision deprivation.

N-acetyl-aspartate (NAA) is an amino acid derivative synthesized in neurons and transported down axons, and it is found exclusively in the neuron and axon [12]. To our knowledge, several studies have correlated the concentration of NAA in brain with the number of neurons measured [8,12-22]. Some reports demonstrated that a decrease of NAA levels is a specific marker for the reduction of neurons and axons, as well as the degeneration including the dysfunction, structural damage and reduced cell size of neurons and axons [8,12-19,22]. In our study, the peak area of NAA and the NAA/Cr ratio in the bilateral visual cortex of monkey 4 in group B were found to be dramatically decreased, while the peak area of NAA slightly decreased and the NAA/Cr ratio clearly decreased in the bilateral visual cortex of monkey 3 in group B than those in group A, which is consistent with previous studies regarding the decrease of NAA and NAA/Cr in unilateral visual cortex of monocular blind rat model [8,22]. So, our results were likely due to the reduction of neurons in the visual cortex of two binocular blind monkeys in group B [8,13-18,22]. In addition, the reduction of axons which transmitted visual inputs to the visual cortex may also lead to the decrease of NAA and NAA/Cr ratio in the visual cortex of two binocular blind monkeys in group B [8,13,14]. What’s more, the degeneration of neurons and axons in the visual cortex can play an important role in resulting in the decrease of NAA and NAA/Cr ratio in visual cortex of two monkeys in group B as well [8,13,14,16,19]. Therefore, in our study the decrease of NAA and NAA/Cr ratio in the visual cortex can indicate the reduction of neurons and axons, as well as the degeneration of neurons and axons in the visual cortex of two binocular blind monkeys in group B.

In contrast to NAA, Ins is found at high concentrations in glial cells [23] and is therefore considered a marker of this cell type [16,24]. A study of blind humans found elevated Ins in the visual cortex compared with normal-sighted individuals, which suggested a proliferation of glial cells, an increase in glial cell size, and/or glial cell participation in the reorganization of the visual cortex in response to vision loss [9]. In our study, the peak area of Ins and the Ins/Cr ratio of monkey 4 slightly increased, which was in accordance with a previous study [9] and suggested that glial cells may take part in visual cortex reorganization in binocular blindness. However, the peak area of Ins and the Ins/Cr ratio of monkey 3 clearly decreased, which may have resulted from individual differences or immune responses to nerve degeneration [25]; therefore, further investigation with a larger sample size is recommended.

The choline (Cho) signal reflects cytosolic choline-containing compounds, 98% of which are phosphocholine and glycerophosphocholine [26]. These compounds can provide Cho for the synthesis of the neurotransmitter acetylcholine (ACh), which is considered as a marker of visual cortex activation [27]. Moreover, according to previous studies in the literature, increased Cho has been observed in conditions with increased numbers of cells, increased membrane synthesis, or increased membrane break down, such as in demyelination [28]. In our results, the ratio of Cho/Cr in the visual cortex of all monkeys in group B dramatically increased compared with group A. We can therefore infer that neurons or glial cells proliferated, that membranes were disrupted, such as in axonal demyelination, and/or that the visual cortex was activated by the remaining sensory systems through cross-modal plasticity. However, a larger sample is needed for further validation.

Glutamate (Glu) and glutamine (Gln) are referred to as Glx. Glu is the major excitatory neurotransmitter in the mammalian central nervous system and is released by neurons in the stimulated state and taken up by surrounding glial cells and converted into Gln [29]. In our study, we found that the peak area of Glx slightly increased in the visual cortex of all monkeys in group B compared with that of group A. This may be due to an imbalance of the Glu-Gln cycle between glutamatergic neurons and astrocytes, and suggests that the neurotransmitter (glutamatergic neurons) may take part in visual cortex reorganization [30]. However, the ratio of Glx/Cr slightly decreased, which indicates that the alteration of Glx maybe unstable or that cross-modal plasticity following vision loss may not lead to the changes of Glx, as was previously reported [31]. To resolve these opposing views, a larger sample size is required.

^1^H-MRS has been widely used to investigate developmental neurobiology in mammalian brains [32,33] to study the metabolite profiles for measuring cross-modal plasticity in response to vision loss or other forms of sensory deprivation [34,35]. There were several limitations in the current study. First, the findings are preliminary, given the limited samples (N=2 for each group), and therefore a larger sample is needed in future studies to improve the reliability of the results. Second, the voxel size used in this study was bigger than the visual cortex for the purpose of ensuring high signal to noise ratio (SNR), which means that signals that were acquired tended to average metabolite concentrations of gray matter, white matter and cerebrospinal fluid. To reduce voxel volume, higher magnetic field MR equipment would be needed. Lastly, we investigated biochemical and metabolic changes in the whole visual cortex, and in future studies we hope to detect these changes in different regions of the visual cortex.

In conclusion, our results demonstrated that biochemical and metabolic alterations occur in the visual cortex of neonatal binocular blind macaque monkeys, as measured with ^1^H-MRS in vivo. These changes were mainly caused by cross-modal plasticity that was associated with the reduction of neurons and axons, the degeneration of neurons and axons, as well as the activation of connections. Such alterations could provide valuable information to investigate the mechanism of cross-modal plasticity in neonatal visual deprivation and other neuroplasticity models.
